# Comparison of Gestational Age Assessment Methods in the Second and Third Trimesters: Evaluating Alternative Approaches Against Ultrasound in Urban Burkina Faso

**DOI:** 10.3390/jcm14051421

**Published:** 2025-02-20

**Authors:** Cheick Ahmed Ouattara, Anderson Compaoré, Lionel Olivier Ouédraogo, Moctar Ouédraogo, Hermann Ouattara, Moussa Coulibaly, Lishi Deng, Zakari Nikiéma, Giles T. Hanley-Cook, Alemayehu Argaw, Lieven Huybregts, Kokeb Tesfamariam Hadush, Carl Lachat, Laeticia Celine Toe, Trenton Dailey-Chwalibóg

**Affiliations:** 1 Agence de Formation de Recherche et d’Expertise en Santé pour l’Afrique (AFRICSanté), Bobo-Dioulasso 01 BP 298, Burkina Faso; ouattaracheickahmed@gmail.com (C.A.O.); discompa4523@gmail.com (A.C.); obmoctar@gmail.com (M.O.); 2 Department of Food Technology, Safety and Health, Faculty of Bioscience Engineering, Ghent University, 9000 Ghent, Belgium; lionel.olivier.ouedraogo@gmail.com (L.O.O.); lishi.deng@ugent.be (L.D.); giles.hanelycook@ugent.be (G.T.H.-C.); alemayehuargaw.alemayehu@ugent.be (A.A.); l.huybregts@cgiar.org (L.H.); kokebtesfamariam.hadush@ugent.be (K.T.H.); carl.lachat@ugent.be (C.L.); laeticiaceline.toe@ugent.be (L.C.T.); 3 Centre Muraz, Bobo-Dioulasso 01 BP 390, Burkina Faso; 4 Centre Médical avec Antenne Chirurgicale de Dô, Bobo-Dioulasso 01 BP 1508, Burkina Faso; houattara@yahoo.fr (H.O.); ouobarr@yahoo.fr (M.C.); 5 Institut Supérieur des Sciences de la Santé, Université Polytechnique de Bobo-Dioulasso, Bobo-Dioulasso 01 BP 1091, Burkina Faso; nikiemazakari@yahoo.fr; 6 Nutrition, Diets, and Health Unit, Department of Food and Nutrition Policy, International Food Policy Research Institute (IFPRI), Washington, DC 20005, USA; 7 Unité Nutrition et Maladies Métaboliques, Institut de Recherche en Sciences de la Santé (IRSS), Bobo-Dioulasso 01 BP 545, Burkina Faso

**Keywords:** gestational age, ultrasound, symphysial fundal height, Dubowitz, DenBalo

## Abstract

**Background**: Accurate determination of gestational age by way of ultrasound is challenging in resource-limited settings like Burkina Faso, leading to the use of alternative methods, though their accuracy and agreement remain poorly established. This practice leads to inadequate risk assessment during pregnancy and failure to identify preterm birth, potentially contributing to high neonatal mortality rates. The purpose of this study was to determine the agreement among alternative methods for gestational age estimation and the Alliance for Maternal and Newborn Health Improvement (AMANHI) method in Burkina Faso. **Methods**: Data were obtained from a prospective cohort study involving pregnant women in the second or third trimester in Bobo-Dioulasso to evaluate the agreement of last menstrual period (LMP), symphysis-fundal height (SFH), Dubowitz, Hadlock, and AMAHNI methods to estimate gestational age. The degree of agreement was assessed using the Bland–Altman method and intraclass correlation coefficients. The AMANHI method, validated for late pregnancy, was used as the reference standard. **Results**: A total of 768 pregnant women were included in the analysis. Plots showed a lack of agreement between the AMANHI method and all other methods, with 95% limits of agreement ranging from −7.6 to +9.8 weeks. Additionally, the incidence of preterm birth was consistently higher when assessed using the alternative methods compared with the AMANHI method. **Conclusions**: The clinical methods (SFH, LMP, Dubowitz) disagree with the ultrasound methods (AMANHI, Hadlock), but the ultrasound methods produce more similar results. The routine application of other methods is likely to result in an overestimation of preterm birth incidence compared with AMANHI. These findings highlight the urgent need to improve access to obstetric ultrasound and to provide comprehensive training in the application of the AMANHI method for accurate late-term gestational age estimation in Burkina Faso.

## 1. Introduction

One of the key Sustainable Development Goals (SDGs) adopted by the United Nations in 2015 is to end preventable deaths of newborns and children under five by 2030. This target urges all countries to reduce the neonatal mortality rate to no more than 12 deaths per 1000 live births [1].

In low- and middle-income countries (LMICs), child mortality rates are disproportionately high during the neonatal period. For instance, in Burkina Faso, the neonatal mortality rate is 23 per 1000 live births, nearly double the global target [2].

Recent studies on the causes of neonatal mortality primarily attribute it to low birth weight, a consequence of preterm birth, or preterm birth itself. In a retrospective cohort study of newborns hospitalized between 1 January 2013 and 31 December 2017, Zoungrana-Yameogo et al. [3] reported a neonatal mortality rate of 22.25 per 1000 person-days in Ouagadougou. Among these cases, 36.05% of the newborns had been admitted due to preterm birth. After adjustment, a birth weight below 1500 g (HRa = 4.13; 95% CI [2.58–6.67]) and the need for resuscitation at birth (HRa 2.62; 95% CI [1.64–4.39]) emerged as significant risk factors for death.

Coulibaly et al. [4] reported a neonatal mortality rate of 53 per 1000 live births in the health districts of Dori and Kaya. After adjustment, preterm birth (HR = 8.0; 95% CI [2.4–26.5]) and low infant birth weight (HR = 0.8; 95% CI [0.7–0.9]) were significantly associated with neonatal death. In such a context, to reduce neonatal mortality, one possible solution is to decrease the incidence of preterm birth by providing adequate prenatal care and care for preterm newborns. This requires the timely identification of potential preterm deliveries and, thus, accurate dating of pregnancies.

There are several methods for estimating gestational age (GA), including the date of the last menstrual period (LMP) reported by the pregnant woman, symphysis-pubis fundal height (SFH), the Dubowitz score, and obstetric ultrasound. While relying on the LMP is a cost-effective approach, numerous studies have highlighted its limitation in accurately estimating gestational age; these inaccuracies can stem from irregularities or individual variations in menstrual cycle length, pre-conceptional amenorrhea following the use of hormonal contraception, implantation bleeding in early pregnancy, and errors in recalling the date of the LMP by the pregnant woman [5,6].

Regarding SFH, the “rule of four”, as outlined in the syllabus for residents of the *French Association of Gynecologists and Obstetricians* and widely applied in clinical practice in Burkina Faso, suggests adding four to SFH measurement in centimeters to estimate GA in weeks (e.g., an SFH of 20 cm is equal to 24 weeks of gestation) [7,8]. This method is very widely used in LMICs, including Burkina Faso, where access to ultrasound remains limited. However, it has several limitations, including inter-operator variability, and does not adequately account for maternal adiposity, intrauterine growth retardation, uterine fibroids, or incorrect fetal presentation [6].

The Dubowitz score, evaluated at birth, is a comprehensive clinical assement tool consisting of 21 criteria designed to estimate GA in weeks. These are divided into two categories: 10 are based on the neurologic maturation of the newborn, such as reflexes ad tone, and 11 are based on external physical characteristics, including skin texture and lanugo [9].

Ultrasound-based estimation of gestational age during the first trimester, up to the 13th week of gestation, is considered the gold standard [10]. Within this timeframe, gestational age assessment using crown–rump length (CRL) measurements provides the highest accuracy. However, beyond 13 weeks, CRL becomes less reliable, necessitating the use of additional biometric parameters to accurately estimate gestational age. The Hadlock algorithm, the standard method for fetal biometry, incorporates multiple measurements, including head circumference, biparietal diameter, abdominal circumference, and femur length, providing a more comprehensive and reliable assessment of gestational age after 13 weeks [6]. However, these standard biometrics do not perform as well in the third trimester [11]. To address this gap, the Alliance for Maternal and Newborn Health Improvement (AMANHI) group has proposed a formula for estimating gestational age using the transcerebellar diameter (TCD) and femur length (FL) to provide accurate gestational age estimation between 24 weeks and 29 weeks 6 days.

Many pregnant women in resource-limited countries do not have access, or only gain late access, to ultrasound, largely due to geographical or financial inaccessibility. For example, a study by Ngowa et al. [5] in Cameroon found that only 23% of pregnant women received obstetric ultrasound in their first trimester. This underscores the significant challenges faced by many women in resource-limited settings, where early pregnancy care is often delayed, making late-pregnancy gestational age estimation methods like AMANHI crucial for improving maternal and neonatal outcomes.

In early 2023, the Description and Comparison of Biological Vulnerability in Small Vulnerable Newborns (SVNs) Versus Healthy Community Controls in Urban Burkina Faso (DenBalo) study was launched (https://clinicaltrials.gov/study/NCT05730569, accessed on 31 May 2024)—the study protocol for which has been published [12]. This cohort study, which includes 70 SVNs and 70 matched, healthy, community controls, aims to apply integrated multi-omics methods to describe and compare the biological vulnerability of SVNs, full-term, appropriate for gestational age (AGA) newborns (>2500 g) in urban areas of Burkina Faso. Pregnant participants were identified during late trimester 2 and trimester 3, and gestational age was estimated using the AMANHI calculation algorithm [6,13]. The data generated by this project will, in the short term, enhance the understanding of biological vulnerability in SVNs. In the medium and long term, the DenBalo study will contribute to the development of targeted strategies and more appropriate care for SVNs in low-income settings [12].

The planned duration of the DenBalo study was determined using preterm birth incidence rates derived from routine statistical data collected at the three Centre de Santé et de Promotion Sociale (CSPS) sites where the study was to be conducted. However, contrary to expectations based on these statistics, no preterm births were reported by the study midwives of the DenBalo project from its launch in January to June 2025.

This unexpected absence of preterm births in the cohort may be attributed to the use AMANHI method, until now untested in Burkina Faso, raising questions about its applicability for estimating gestational age in the second and third trimester in this context.

Thus, the primary objectives of this study were twofold: First, this study aimed to compare the accuracy gestational age estimation methods, including LMP, SFH, the Hadlock algorithm, against the AMANHI method in Burkina Faso. Second, this study aimed to estimate and compare the incidence of preterm birth that would have been reported in the DenBalo cohort using each of these methods.

## 2. Materials and Methods

### 2.1. Study Area and Population

GA data were collected from the DenBalo cohort study conducted between January 2023 and February 2024 in four health centers, including three (CSPS) and one Centre Médical avec Antenne Chirurgicale (CMA), in the Dô Health District, Bobo-Dioulasso, Burkina Faso. This study was approved by the Commissie voor Medische Ethiek (CME) of Ghent University Hospital (protocol code: ONZ-2022-0500 and date of 29 November 2022) and the Comité d’Éthique Institutionnel de la Recherche En Sciences de la Santé (CEIRES) of the Institut de Recherche en Sciences de la Santé (IRSS) (protocol code A40-2022/CEIRES and date of 19 October 2022) and aim to apply integrated multi-omics methods to characterize the fundamental biology of maternal and newborn health vulnerabilities linked to delivery term and birth weight. The study population consisted of pregnant women in the early third trimester attending regular antenatal consultations (ANC) at one of the three CSPS.

### 2.2. Enrollment and Assessments

The enrollment process for pregnant women consisted of two stages. First, an initial screening was conducted at the CSPS during the prenatal visits based on the measurement of the SFH. Each pregnant woman underwent dual SFH measurements, taken from the highest point of the uterus (fundus) and the top of the pubic symphysis, using a non-elastic measuring tape by midwives trained specifically for this procedure. Pregnant women whose average SFH fell between 24 and 27 cm were considered pre-eligible, and written informed consent was obtained.

The second stage involved estimating gestational age using ultrasound at the CMA. A trained gynecologist performed the ultrasound using a portable machine (Sonosite Edge II, FujiFilm, MKT02792). Measurements included head circumference, biparietal diameter, and abdominal circumference (each taken once), as well as TCD and FL, both measured in duplicate. If repeated measurements showed discrepancies exceeding ±1 week of gestational age (i.e., >1.8 mm for TCD or >0.24 mm for FL), a third measurement was required. Gynecologists evaluated image quality based on predefined criteria (e.g., zoom level, correct plane freezing, and caliper placement) and ranked the images in descending order, from best to worst [i.e., first, second (and possibly third)]. The average measurements of the two best-quality images were used to determine gestational age.

Following the ultrasound, singleton pregnancies between 24 and 30 weeks of gestation were deemed definitively eligible for this study. Pregnancies with detected organ malformations or structural abnormalities identified during ultrasound were excluded from this study. Eligible women were monitored until delivery, with an additional prenatal visit at 36 weeks of gestation, during which the SFH measurement was repeated.

GA at birth was assessed using the following methods: (i) LMP [14], (ii) SFH [7,8] between the 24th and 30th weeks of gestation, (iii) SFH at the 36th week, (iv) Hadlock formula [15], (v) AMANHI formula [6], and (vi) Dubowitz score [9] (Table 1). We calculated the gestational age at delivery by incorporating the time interval between the obstetric ultrasound date and the date of birth (d), which was converted into weeks. This interval was added to the gestational age at the screening ultrasound (AMANHI, Hadlock).

### 2.3. Data Management and Statistical Analysis

Data were collected using forms created in Survey Solutions and deployed on tablets. The data downloaded from the server were processed to exclude outlier values below the 5th percentile or above the 95th percentile.

Agreement of the methods was assessed using Bland–Altman plots [16] and intra-class correlation coefficient (ICC) [17]. Pairwise comparisons were performed as follows: (i) SFH at inclusion versus AMANHI, (ii) SFH at 36 weeks of gestation versus AMANHI, (iii) Hadlock versus AMANHI, and (iv) Dubowitz score versus AMANHI. In the Bland–Altman analysis, pairwise comparisons were conducted by calculating the mean [i.e., (measurement by AMANHI + measurement of another method)/2] and the difference [(measurement by AMANHI—measurement of another method)] for each study participant. These values were plotted, with the mean on the horizontal axis and the differences on the vertical axis. The normality of the distribution of the difference was tested using the Shapiro–Wilk test [18,19]. BA test if the mean difference is significantly different from zero and if the limits of agreement are acceptable. A positive value indicates that the same parameter obtained the second time resulted in a smaller measurement. By contrast, a negative value indicates that the same parameter obtained the second time resulted in a larger measurement [20].

The ICC was assessed using a two-way mixed effects model to estimate the variance attributed to the random subject effect and residual variance [21]. ICC agreement was interpreted as follows: poor (≤0.40), fair (0.40–0.59), good (0.60–0.74), and excellent (≥0.75) [18].

For each method, gestational age was determined in days at the time of screening. To calculate the gestational age at birth, the number of days between the screening date and the neonate’s birth was added to the gestational age assessed at screening. Newborns with gestational age at birth ≥37 weeks were classified as “term”, while those with gestational age at birth less than 37 weeks were categorized as “pre-term”. The incidence of preterm birth was calculated, and Cohen’s Kappa coefficient (κ) was used to quantify agreement in preterm classification. The interpretation of κ values is as follows: no agreement (≤0), none to slight (0.01–0.20), fair (0.21–0.40), moderate (0.41–0.60), good (0.61–0.80), and perfect (0.81–1.00) [22]. Data analysis was performed using R (4.3.3).

## 3. Results

**Figure 1:**
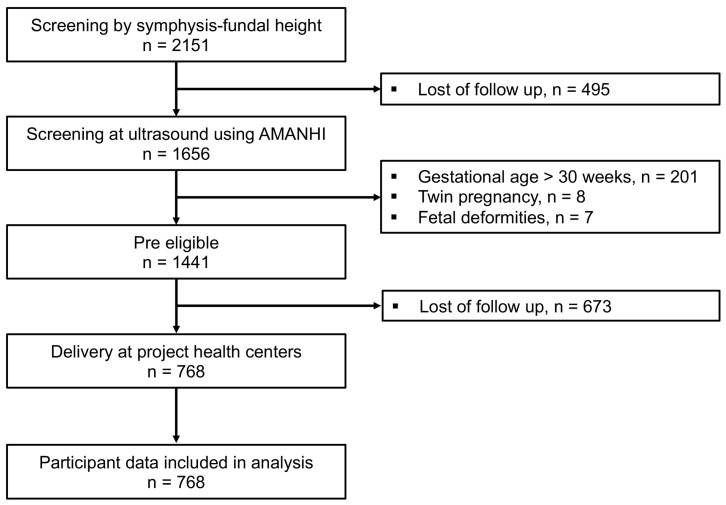
Study flowchart.

During the study period, 768 pregnant women met the eligibility criteria and were included in the analysis (Figure 1).

Upon enrollment, the cohort had a mean ± standard deviation (SD) age of 25.92 ± 6.15 years, a mean height of 162 ± 6 cm, and a mean weight of 68.31 ± 12.96 kg (Table 2). Notably, one-fourth (24.30%) of participants reported being illiterate, while a mere 2.2% possessed a higher educational attainment. On average, participants attended 2.36 ± 0.94 antenatal visits (ANV). Furthermore, the mean birth weight of newborns was 3010 ± 420 g, with 8.89% of them classified as having a low birth weight (LBW).

The LMP was known by only 4% (n = 31) of pregnant women. GA data at 36 weeks (n = 186) were missing due to some participant not attending their 36-week appointments. Missing data for the Dubowitz score resulted from deliveries occurring outside the recruitment sites or those not attended by the project’s midwives. Descriptive statistics for each of the methods are shown in Table 3. Among the methods, SFH at 24–30 exhibited the widest range of estimated GA, followed by the Dubowitz score.

### 3.1. Agreement Among Gestational Age Assessment Methods

**Figure 2:**
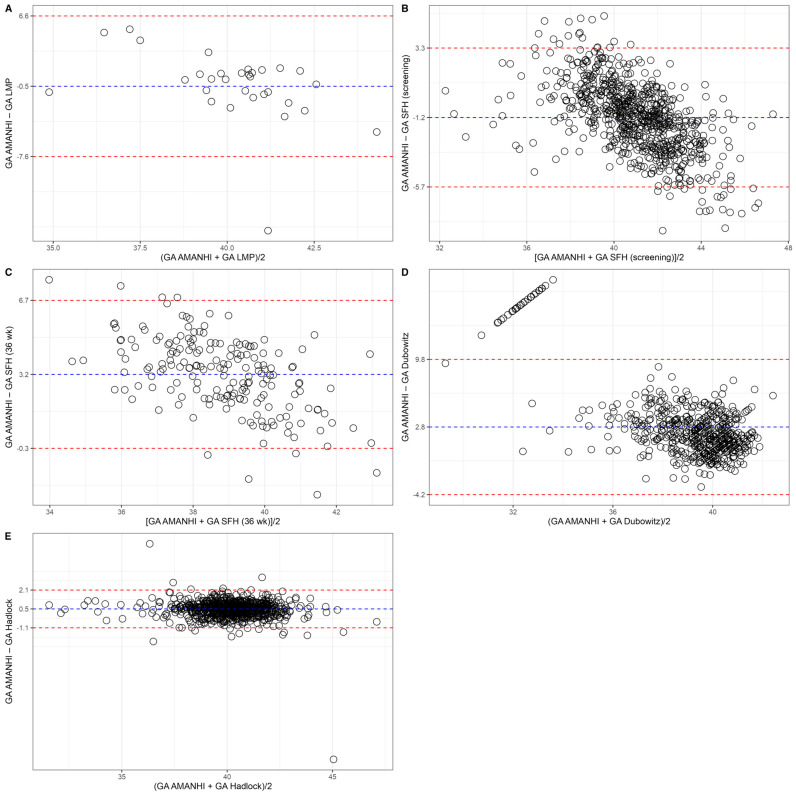
Comparisons of gestational age estimation between the Alliance for Maternal and Newborn Health Improvement algorithm and (**A**) last menstrual period, (**B**) symphysis-pubis fundal height at first screening, (**C**) symphysis-pubis fundal height at first 36th gestational week (**D**) Dubowitz score, and (**E**) Hadlock algorithm.

Compared with the AMANHI method, both the LMP and SFH approaches (measured between the 24th and 30th weeks of gestation) tended to overestimate gestational age by an average of 0.5 weeks (95% limit of agreement: −7.6 to +6.6 weeks) and 1.2 weeks (−5.7 to +3.3 weeks), respectively (Figure 2).

In contrast, SFH measurements at the 36th week of gestation, the Dubowitz score, and the Hadlock method tended to underestimate gestational age. Specifically, gestational age was underestimated on average by 3.2 weeks (−0.3 to +6.7 weeks), 2.8 weeks (−4.2 to +9.8 weeks), and 0.5 weeks (1.1 to +2.1 weeks), respectively.

With the exception of the Hadlock method (ICC = 0.860), which demonstrated excellent intra-method concordance, the ICC values for other methods indicated poor agreement (Table 4).

### 3.2. Preterm Incidence Estimates

According to the AMANHI method, the incidence of preterm birth was 2.80%. In comparison, all other methods overestimate preterm rates, ranging from 3.86% for the Hadlock method to 43.96% for SFH at the 36th week (Table 5).

This discrepancy was further confirmed using the Kappa coefficient, which indicated poor agreement between the AMANHI method and other approaches, including the LMP method (κ = 0.271, *p* = 0.106), SFH at 24–30 weeks (κ = 0.400, *p* = 0.001), SFH at 36 weeks (κ = 0.027, *p* = 113), and the Dubowitz score (κ = 0.120, *p* = 0.001). In contrast, the Hadlock method showed strong agreement with the AMANHI method (κ = 0.722, *p* = 0.001).

## 4. Discussion

The aim of this study was to assess the accuracy of GA estimation methods, particularly in the urban context in Burkina Faso. The finding reveals significant variability in GA estimation when comparing routinely used methods—such as LMP, SFH, or the Hadlock method during pregnancy and Dubowitz score at delivery—to the AMANHI method. All of these methods overestimate preterm birth incidence in our cohort.

In LMICs, SFH is commonly used to assess GA due to limited access to more accurate methods, such as ultrasound. Given the higher incidence of pregnancy-related complications and fetal growth restriction in these settings, it is essential to validate the accuracy of SFH locally [8]. Previous studies have consistently highlighted the limitations of utilizing LMP or SFH for estimating gestational age.

Ngowa et al. [5] in Cameroon and Rosenberg et al. [23] in Ghana reported a high frequency of discrepancies (42.07% and 39.2%, respectively) between gestational age determined by LMP and early obstetric ultrasound. Factors significantly associated with these discrepancies included reliance on memory for LMP recall, irregular menstrual cycles, and uncertainty about the reported LMP. In our study, the majority of participants could not recall their LMP. This is likely due to the recruitment of women in their second and third trimesters, a period when recollection of LMP becomes increasingly difficult and unreliable.

The accuracy of SFH can be affected by various factors, including multiple pregnancies, maternal size, intrauterine growth retardation, fetal position, and measurements collected beyond the second trimester [24].

Jehan et al. [14] reported in an urban setting in Pakistan that SFH measurements were comparable in accuracy to ultrasound using the Hadlock method among 1128 women screened between 20 and 26 weeks of pregnancy. In 75% of cases, the GAs estimated using the SFH method were within 7 days of those estimated by the Hadlock method. Similarly, in 91% of cases, SFH estimates were within 14 days of the Hadlock estimates. However, in 6.7% of cases, the SFH method resulted in GA differences exceeding 14 days compared with the Hadlock method [14].

Our study demonstrates that using SFH to estimate GA significantly overestimates the incidence of preterm birth. Similarly, Jehan et al. reported cases of misclassification of newborns with routine SFH estimates. They found that 31.7% of newborns classified as preterm by ultrasound-based gestational age were classified as term by SFH, while 15% of newborns classified as full-term by ultrasound were classified as preterm by the SFH. Inaccurate estimation of gestational age can have wide-ranging consequences, including mismanagement of antenatal care [25], reduced likelihood of delivery at a health facility [26], and diminished effectiveness of public health, neonatal and maternal health programs due to the misclassification of preterm and post-term births [25]. Furthermore, it can compromise risk assessment and surveillance of adverse perinatal outcomes.

More recently, Welan et al. [8], in a recent systematic review, emphasized that SFH should not be relied upon as the sole method for determining GA. Their findings indicated that SFH estimates of GA were considerably less accurate compared with ultrasound-confirmed dating, with a wide margin of error. Consequently, it is imperative to reduce or even eliminate reliance on SFH for GA estimation in both clinical antenatal care and research. Instead, efforts should focus on expanding access to ultrasound services and providing comprehensive training in advanced ultrasonography methodologies, such as the AMANHI approach, particularly in LMICs. By broadening access to ultrasound, we can ensure that pregnancy monitoring and the implementation of life-saving interventions are informed by precise estimates of GA.

In their study comparing various methods for estimating gestational age based on the date of the LMP in Cameroon, Sunjoh et al. [27] reported that the Dubowitz method demonstrated the highest validity, achieving a 93% agreement within ±2 weeks of gestational based on LMP dates. Similarly, Feresu et al. [28] reported in Zimbabwe that the Dubowitz method proved to be a valuable predictor of gestational age, effectively distinguishing between term and preterm term and preterm infants when LMP was used as a reference. These findings highlight the superior performance of the Dubowitz score compared with SFH while acknowledging a two-week margin of error, consistent with the average difference of 2.3 weeks in our study.

We found no studies comparing the AMANHI method to the other gestational age estimation methods assessed in this study. The AMANHI method has shown great promise in improving gestational age estimation during the second and third trimesters of pregnancy [6]. Our findings indicate that the methods routinely used in Burkina Faso are not suitable substitutes for the AMANHI method. This highlights the critical need to integrate the AMANHI method as a standard approach for gestational age estimation during the later stages of pregnancy.

Achieving this integration requires expanding access to obstetric ultrasound and providing targeted training for clinicians in the application of the AMANHI method. By implementing these measures, on one hand, we can ensure more accurate gestational age estimation, which is essential for better risk assessment and the delivery of appropriate care to pregnant women and newborns. On the other hand AMANHI has the advantage that the TCD and FL might be technically easier to measure more reliably by personnel with minimal training or experience.

Our study has some limitations. Due to the study setting, where women typically attend their first antenatal consultation after the first trimester of gestation, we had to rely on ultrasound estimates conducted in the second and third trimesters. Previous studies have indicated that such late estimates can be influenced by restricted fetal growth prior to the measurements, potentially leading to an underestimation of gestational age [8]. Among late ultrasound dating methods, we considered the gestational age obtained using the AMANHI method as the reference. According to the literature, this method provides the most reliable estimation of gestational age in this context [6]. However, we acknowledge that the use of AMANHI as the reference standard is a limitation of our study. Additional validation is needed in a sample with known GA (such as in vitro fertilization pregnancy) or at least with first-trimester crown–rump length measurement to test whether AMANHI is really more accurate than Hadlock.

Furthermore, the limited number of participants who were able to recall the date of their last menstrual period might have affected the accuracy assessment of this method. However, this reflects the practical challenges of routinely relying on LMP in this specific context.

## 5. Conclusions

The discrepancy in preterm birth incidence observed in our cohort compared with the data reported by health centers is likely attributable to classification errors in the statistical data used for forecasting. These errors stem from the gestational age estimation methods currently employed in health facilities across Burkina Faso.

Expanding access to ultrasound for pregnant women, coupled with training physicians to accurately measure the transcerebellar diameter via ultrasound, will enable more precise gestational age determination in late pregnancy using the AMANHI method. This advancement could significantly improve the identification of small vulnerable newborns and enhance neonatal care in resource-constrained settings, like Burkina Faso. Furthermore, the findings from our study contribute to a deeper understanding of gestational age assessment in low-resource contexts, providing a foundation for targeted public health programming to improve and optimize neonatal care and outcomes.

## Figures and Tables

**Table 1:** Methods of gestational age, in weeks.

Methods	Formulae
Last menstrual period	(Date of birth−Date of LMP)7
Symphysis-fundal height (*all parameters measured in cm*) [7,8]	$\left(SFH+4\right)$
Hadlock (*all parameters measured in cm*) [15]	$\left(\left(10.61+\left(0.175\times BPD\times FL\right)+\left(0.297\times AC)\right)\right)+\left(0.71\times FL\right)\right)$
AMANHI (*all parameters measured in mm*) [6]	e0.3825021×lnTCD+0.3321277×ln⁡FL+2.634167
Dubowitz score [9]	0.2642×DS+24.595

LMP: Last menstrual period; SFH: Symphysis-fundal height at screening; TCD: Trans cerebellar diameter at screening; FL: Femur length at screening; BPD: Biparietal diameter at screening; AC: abdominal circumference at screening; DS: Dubowitz score at birth; AMANHI: Alliance for Maternal and Newborn Health Improvement.

**Table 2:** Characteristics of mothers/infants (n = 768).

Characteristic	Summary Statistic ^1^
Age (years)	25.92 ± 6.15
Age < 20 (years)	120 (15.65%)
Illiterate	173 (24.30%)
Remunerative activity	323 (45.37%)
Parity	2.34 ± 1.44
Primiparity	181 (22.15%)
Height (cm)	162 ± 6.00
Weight (kg)	68.31 ± 12.96
MUAC ^1^ (cm)	26.99 ± 3.34
Antenatal visit	2.36 ± 0.94
AMANHI Gestational age at delivery (weeks)	40.39 ± 1.67
Birth weight (g)	3010 ± 420
Low birth weight (<2500 g)	67 (8.89%)

^1^ Reported as mean ± SD or n (%). MUAC, mid-upper arm circumference. AMANHI, Alliance for Maternal and Newborn Health Improvement.

**Table 3:** Descriptive statistics of each gestational age assessment method.

Methods	n	Minimum	Maximum	Mean	Median
Last menstrual period	31	34	49	40	40
Symphysis-Fundal Height (24–30th weeks)	768	32	50	42	42
Symphysis-Fundal Height (36th week)	186	30	44	37	37
Hadlock	768	31	51	40	40
Dubowitz	582	25	42	39	38
AMANHI	768	32	47	40	40

AMANHI, Alliance for MAternal and Newborn Health Improvement.

**Table 4:** Intraclass correlation coefficients of gestational age assessment methods.

Comparison	ICC ^1^	Lower CI ^1^	Upper CI ^1^	*p* Value
LMP ^1^ vs. AMANHI	0.049	−0.312	0.395	0.396
SFH ^1^ at 24–30th week vs. AMANHI	0.474	0.273	0.612	0.001
SFH at 36th week vs. AMANHI	0.235	−0.088	0.547	0.146
Hadlock vs. AMANHI	0.860	0.724	0.917	0.001
Dubowitz vs. AMANHI	0.122	−0.008	0.242	0.033

^1^ LMP: Last menstrual period. ICC: intraclass correlation coefficient. CI: confidence interval. SFH: symphyseal fundus height, AMANHI, Alliance for MAternal and Newborn Health Improvement.

**Table 5:** Proportion of prematurity assessment by different methods.

Methods	Subjects	Preterm % (n)
AMANHI	768	2.9 (22)
Hadlock	768	3.9 (30)
Symphyseal fundus height at 24–30th week	768	4.6 (35)
Last menstrual period	31	12.9 (4)
Dubowitz	582	25.1 (146)
Symphyseal fundus height at 36th week	186	44.6 (83)

AMANHI, Alliance for MAternal and Newborn Health Improvement.

## Data Availability

Due to the sensitive nature of the data, access will be granted through a data-sharing agreement. For inquiries, please contact trenton.daileychwalibog@ugent.be.
